# Stigmasterol attenuates hepatic steatosis in rats by strengthening the intestinal barrier and improving bile acid metabolism

**DOI:** 10.1038/s41538-022-00156-0

**Published:** 2022-08-27

**Authors:** Yaxin Zhang, Yuyan Gu, Jing Jiang, Xiaobing Cui, Saibo Cheng, Linling Liu, Zhiyong Huang, Rongxin Liao, Peng Zhao, Jieying Yu, Jing Wang, Yuhua Jia, Wen Jin, Fenghua Zhou

**Affiliations:** 1 Third Level Research Laboratory of State Administration of Traditional Chinese Medicine, School of Traditional Chinese Medicine, Southern Medical University, Guangzhou, Guangdong 510515 China; 2 Integrated Hospital of Traditional Chinese Medicine, Southern Medical University, Guangzhou, Guangdong 510315 China; 3 School of Traditional Chinese Medicine, Southern Medical University, Guangzhou, Guangdong 510515 China; 4 Department of Cardiology, Integrated Hospital of Traditional Chinese Medicine, Southern Medical University, Guangzhou, Guangdong 510315 China; 5 Department of Otolaryngology, The Third Affiliated Hospital of Southern Medical University, Guangzhou, Guangdong 510630 China; 6 Center of TCM Preventive Treatment, Integrated Hospital of Traditional Chinese Medicine, Southern Medical University, Guangzhou, Guangdong 510315 China; 7 Department of Cardiac Intensive Care Unit, Cardiovascular Hospital, Guangdong Second Provincial General Hospital, Guangzhou, Guangdong 510317 China

**Keywords:** Obesity, Pathogens, Dyslipidaemias

## Abstract

Stigmasterol (ST) has been shown to improve both lipid and bile acid (BA) metabolism. However, the mechanism(s) by which ST prevents dyslipidemia via BA metabolism, and the potential involvement of other regulatory mechanisms, remains unclear. Here, we found that ST treatment effectively alleviates lipid metabolism disorder induced by a high-fat diet (HFD). Moreover, we also show that fecal microbiota transplantation from ST-treated rats displays similar protective effects in rats fed on an HFD. Our data confirm that the gut microbiota plays a key role in attenuating HFD-induced fat deposition and metabolic disorders. In particular, ST reverses HFD-induced gut microbiota dysbiosis in rats by reducing the relative abundance of *Erysipelotrichaceae* and *Allobaculum* bacteria in the gut. In addition, ST treatment also modifies the serum and fecal BA metabolome profiles in rats, especially in CYP7A1 mediated BA metabolic pathways. Furthermore, chenodeoxycholic acid combined with ST improves the therapeutic effects in HFD-induced dyslipidemia and hepatic steatosis. In addition, this treatment strategy also alters BA metabolism profiles via the CYP7A1 pathway and gut microbiota. Taken together, ST exerts beneficial effects against HFD-induced hyperlipidemia and obesity with the underlying mechanism being partially related to both the reprogramming of the intestinal microbiota and metabolism of BAs in enterohepatic circulation. This study provides a theoretical basis for further study of the anti-obesity effects of ST and consideration of the gut microbiota as a potential target for the treatment of HFD-induced dyslipidemia.

## Introduction

As a multi-factorial disease, hyperlipidemia is becoming a non-negligible health problem and is one of the main causes of cardiovascular diseases (CVD), which is the leading cause of death worldwide^1–3^. Synthesis and excretion of bile acids (BAs), which mainly occurs in the liver, is closely related to hyperlipidemia^4^. Since the accumulation of lipids in the blood and liver is the main cause of hyperlipidemia, the recovery of blood lipids, especially the metabolism of cholesterol and BAs, is the principal strategy for treating hyperlipidemia. The classical neutral pathway and alternative or acidic pathway are two methods of converting cholesterol into BAs. BA metabolism is regulated by many factors, including BA receptors, transporters, genes, and proteins associated with BA entering the liver circulation. Farnesoid X receptor (FXR, also known as NR1H4) is a key receptor for regulating the balance of BAs, and chenodeoxycholic acid (CDCA) is the most effective ligand for FXR^5,6^.

BAs have become important pleiotropic signal metabolites, which are involved in regulating metabolism and inflammation via interactions with the microflora and host receptors^7^. Intestinal microflora can influence BA metabolism by maintaining the BA pool through several BA modifications in processes such as deconjugation and dehydroxylation^8,9^. Microbes can convert cholic acid (CA) and CDCA into dicarboxylic acid (DCA) and tricarboxylic acid (TCA) in the large intestine by dehydrogenation of BA 7-dehydroxylase. Conjugated BAs produced in the liver are deconjugated in the ileum by the microbial enzyme, bile salt hydrolase, in a modification process that increases BA activity^10^. Additionally, the intestinal barrier plays an important role in maintaining the stability of the intestinal environment, as well as maintaining the symbiotic balance of the intestinal microflora, which is implicated in lipid production and metabolism^11^. The mucus barrier formed by goblet cell secretions is the first intestinal barrier that is in the most direct contact with the external environment^12^. Pathophysiological changes that occur in the luminal environment of the intestines due to a HFD, are closely associated with minimal inflammation. The gut–liver axis is implicated in this process through disturbances in the integrity of the intestinal mucosa.

Phytosterols, naturally occurring compounds which are similar to cholesterol in their structure and function^13^, reduce intestinal cholesterol absorption and help to lower low-density lipoprotein cholesterol (LDL-C) levels^14^. Consequently, phytosterols are Food and Drug Administration approved cholesterol-lowering agents^15^. Phytosterols have also been proven to have immunomodulatory properties^16^. There is increasing evidence showing that stigmasterol (ST), a type of phytosterol, induces therapeutic effects in hyperlipidemia and obesity^17–19^. Moreover, ST significantly improves fatty liver and metabolic abnormalities that are caused by a high-fat Western diet^17^. However, its potential mechanism(s) remain undefined. Interestingly, ST also attenuates inflammatory bowel disease, possibly by activating the butyrate-PPARγ axis to restore immune balance in Treg/Th17 cells, leading to improved gut microbiota disequilibrium^20^.

We hypothesized that ST treatment might regulate intestinal barrier function and lead to the change of cholesterol metabolism, which given its involvement in BA metabolism. To test this hypothesis, we employed liquid chromatography-mass spectrometry (LC-MS) to analyze the serum and fecal BA spectra. To further understand the potential mechanism(s) of ST-induced improvements in hyperlipidemia and BA metabolism, we also examined the expression of genes related to cholesterol and BA metabolism in a model of hyperlipidemia in Sprague–Dawley (SD) rats. Furthermore, we used fecal microbiota transplantation (FMT) to confirm the function of the gut microbiota in HFD-fed rats. Herein, we provide novel evidence in support of ST as a potential therapy for hyperlipidemia.

## Results

### ST ameliorates hyperlipemia and hepatic steatosis in HFD-fed rats

**Fig. 1:**
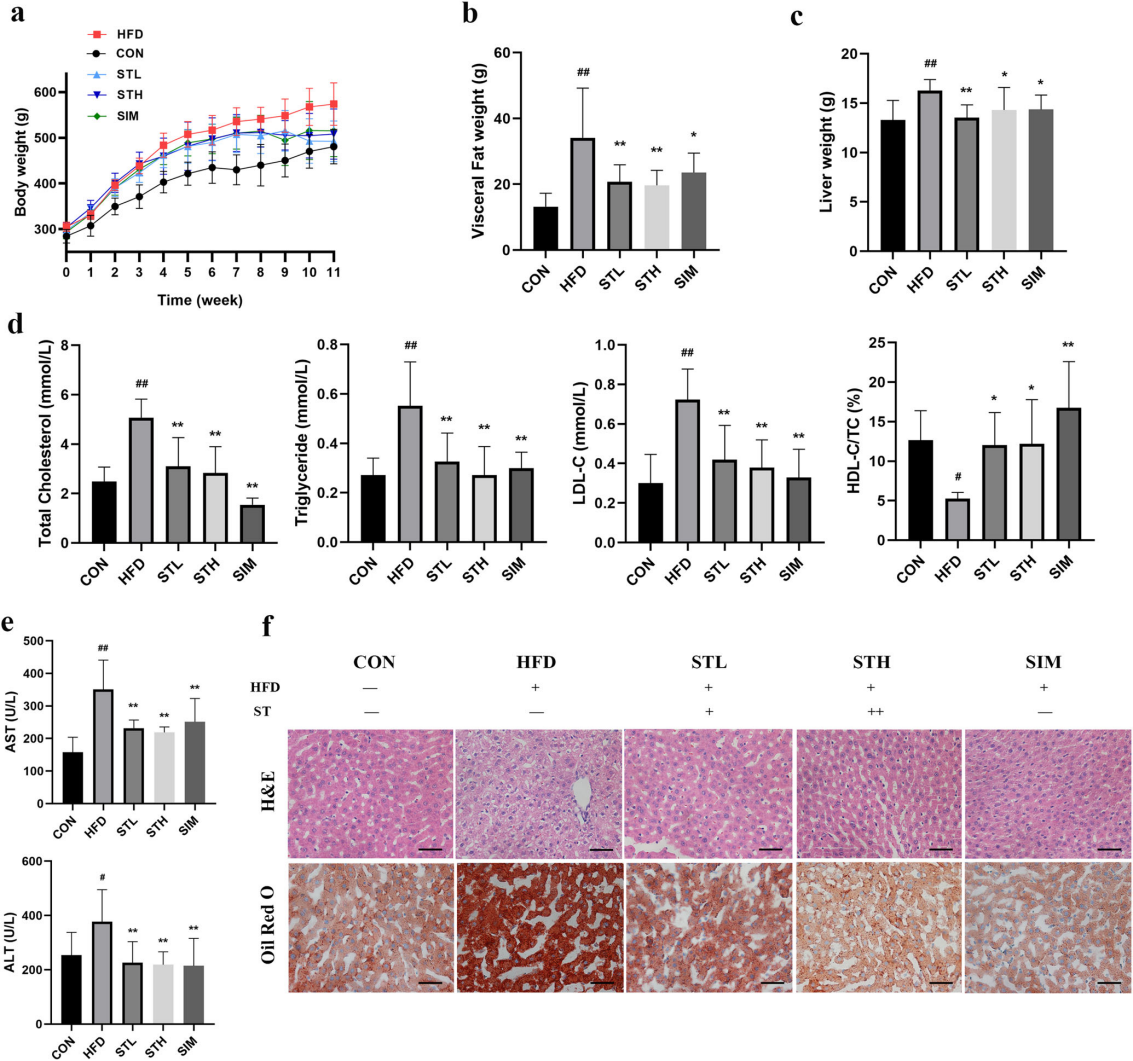
Stigmasterol (ST) attenuated HFD-induced hyperlipemia and hepatic steatosis in HFD fed rats. **a** Changes in the body weight of rats over 11 weeks. At week 11, multiple obesity-related parameters were recorded for the five groups of rats receiving HFD, HFD + low-dose stigmasterol (STL), HFD + high-dose stigmasterol (STH), HFD + simvastatin group (SIM), control diet (CON), respectively. The parameters of rats included: **b** viseral fat weight; **c** liver weight; **d** serum levels of lipid (TC, TG, LDL-C, HDL-C/TC); **e** serum levels of liver function (AST and ALT); **f** H&E staining and ORO staining of liver, Scale bar: 50 μm. Data are expressed as mean ± standard deviation. *n* = 8. One-way ANOVA was used to analyze statistical differences; compared with control group, ^#^*P* < 0.05, ^##^*P* < 0.01; compared with HFD group, ^*^P < 0.05, ^**^P < 0.01.

To study the effects of ST on hyperlipidemia, SD rats were fed a HFD for 11 weeks. During the 11-week experiment, the weight of all experimental groups was recorded weekly. As expected, the HFD resulted in increased body weight compared to the control group which received a normal diet (*P* < 0.01). Importantly, daily treatment with ST significantly reduced weight gain in HFD-fed rats (Fig. 1a). In addition, ST also lead to a reduction in the weight of visceral fat and liver (Fig. 1b, c). Compared to the control group, the HFD group showed significantly increased levels of serum TC, TG, and LDL-C by 2.04-, 2.03-, and 2.40-fold, respectively, which were all significantly reduced upon treatment with ST (*P* < 0.01; Fig. 1d).

Compared to the control group, the levels of serum AST and ALT in HFD group were significantly upregulated by 2.22- and 1.49-fold, respectively. ST significantly reduced these levels by 0.62- and 0.58-fold, respectively (Fig. 1e). ST treatment also had a positive impact on hepatic steatosis as evidenced by hematoxylin and eosin (H&E) and Oil Red O (ORO) staining. Compared to the control group, liver sections from HFD-fed rats showed obvious fat deposition, which was significantly improved after ST treatment (Fig. 1f). Moreover, the high-dose group showed a more obvious improvement in liver lipid deposition.

### ST improves intestinal barrier function in HFD-fed rats

**Fig. 2:**
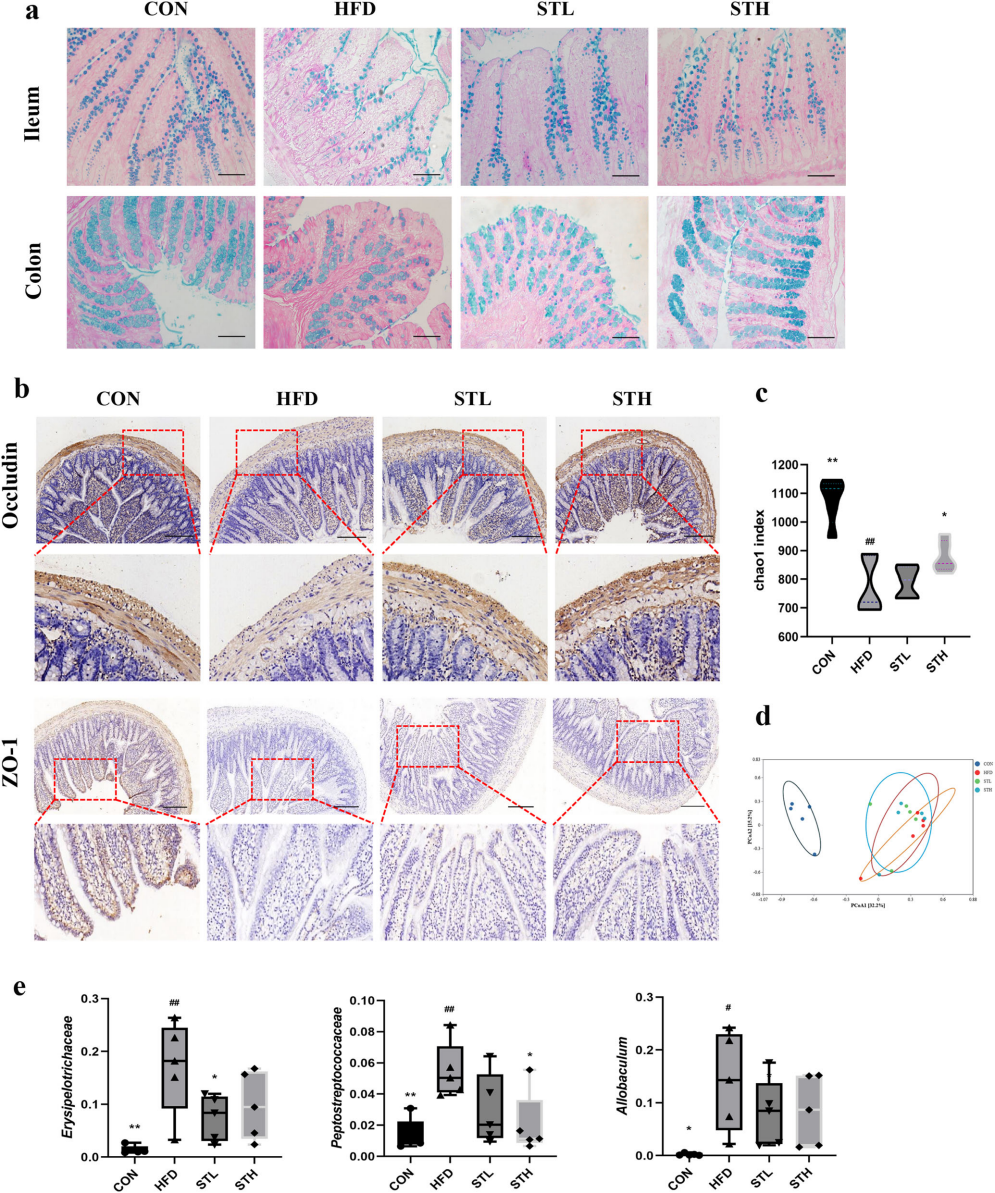
ST improved intestinal barrier function in HFD fed rats. **a** AB-PAS staining of ileum and colon, Scale bar: 100 μm; **b** immunohistochemistry of occludin and ZO-1 protein in ileum, Scale bar: 200 μm; **c** the α-diversity index (chao1) of gut microbiota; **d** the β-diversity analysis (PCoA) of gut microbiota; **e** relative abundance of biomarker genra in family level and genus level. Data are expressed as mean ± standard deviation. One-way ANOVA was used to analyze statistical differences; compared with control group, ^#^*P* < 0.05, ^##^*P* < 0.01; compared with HFD group, ^*^P < 0.05, ^**^P < 0.01.

The intestinal barrier mainly consists of a mechanical-, immune-, chemical-, and biological barrier. The mucosal function of the intestinal barrier depends on the protective mucus layer which is produced by goblet cells. To determine whether ST can improve intestinal barrier function, we first evaluated the intestinal barrier function of SD rats by H&E and AB-PAS staining. We found that the number of goblet cells clearly decreased in the HFD group compared to the control group (Fig. 2a). Following ST treatment, the number of goblet cells increased. This is important as it may promote improvement of mucus secretion disorders and flora disorders. Further, compared to the HFD group, ST treatment also increased the protein expression of occludin and ZO-1 in the ileum using IHC (Fig. 2b).

To understand whether these ST-induced effects were mediated by the recombination of intestinal microflora, we examined bacterial flora in fecal samples from experimental groups (i.e., control group, HFD group, ST-L group, and ST-H group) by 16s rDNA gene sequence analyses. However, compared to HFD-fed rats, the α-diversity of the ST group was significantly increased as measured by the Chao 1 index (Fig. 2c). Principal component analysis (PCoA) showed that the overall structure of the intestinal flora in HFD groups was similar. In this study, compared to HFD group, ST treatment also reduced levels of *Erysipelotrichaceae* (16.71% versus 9.77%) and *Peptostreptococcaceae* (5.47% versus 2.03%) (*P* < 0.05) bacteria, which may be helpful in the prevention of HFD. Incredibly, these data show that ST changes the structure and composition of the intestinal microflora. However, it is not clear whether intestinal microbes play a major role in intestinal barrier function. Taken together, these results show that ST treatment improves intestinal barrier function in HFD-fed rats.

### ST alters serum and fecal BA profiles in HFD-fed rats

**Fig. 3:**
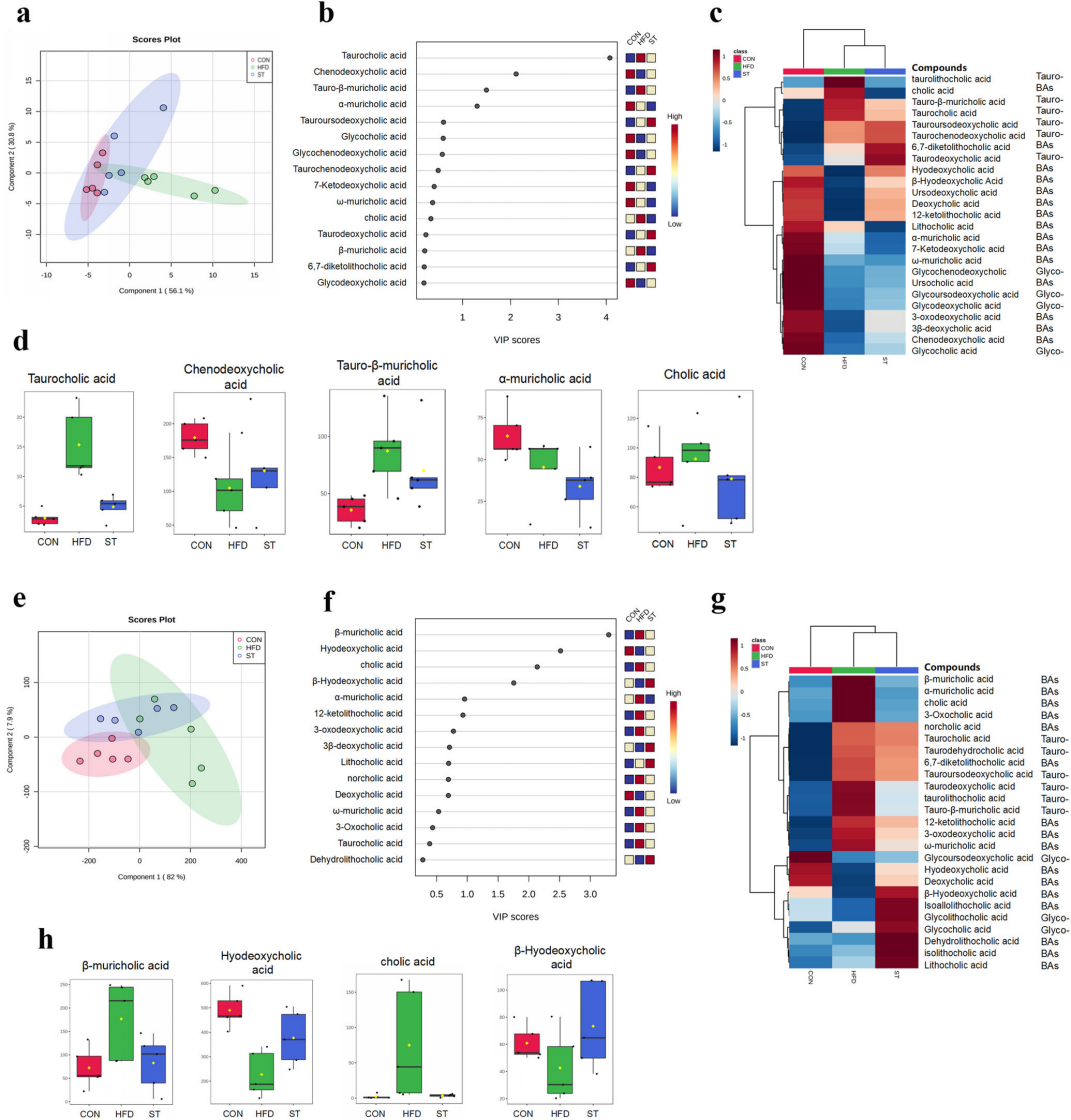
ST altered serum and fecal BA profiles in HFD fed rats. **a** PLS-DA scores plots in serum among the groups; **b** variable importance in projection (VIP) scores were identified by PLS-DA; **c** hierarchical clustering heatmaps of the top 25 BAs contents in serum among the groups; **d** the levels of biomarker BAs in serum among groups; **e** PLS-DA scores plots in feces among the groups; **f** VIP scores were identified by PLS-DA; **g** hierarchical clustering heatmaps of the top 25 BAs contents in feces among the groups; **h** the levels of biomarker BAs in feces among groups.

BAs are metabolites of cholesterol metabolism, which is secreted into the intestine through the bile tree. These BAs control metabolism within and outside of the liver, especially for energy balance^21^. Importantly, secreted BAs are processed (i.e., combined) by the gut microbiota into secondary BAs and affect the growth of BA-metabolizing bacteria. To investigate the change of BAs, we measured serum and fecal BAs (including primary and secondary BAs) among rats in the control, HFD, and STH groups using LC/MS. PLS-DA was performed to determine the clustering trend of the data. We found that the ST group was sightly different from HFD group (Fig. 3a). According to standard analysis of VIP score > 1, we found that TCA contributes most among those BAs with a marked increase in the HFD group and a decrease in the ST group (Fig. 3b). Notably, compared to the HFD group, the CDCA in the ST group was significantly higher. We also found that serum Tauro-β-muricholic acid (T-β-MCA) and CA levels decreased in the in ST group compared to HFD-fed rats (Fig. 3c, d).

Interestingly, we found that fecal BA structures differed compared to serum BAs. Among these BAs, β-hydrochloric acid (β-HCA) contributed the most to the change of the structure, which increased significantly in the HFD group and decreased in the ST group (Fig. 3f, h). Fecal levels of the hyodeoxycholic acid (HDCA) and β-Hyodeoxycholic Acid (β-HDCA) were upregulated while CA levels decreased in the ST group compared to the HFD group (Fig. 3h). In addition, levels of fecal α-muricholic acid (α-MCA), β-MCA, taurolithocholic acid (TLCA), taurodeoxycholic acid (TDCA) were lower in the control and ST groups compared to the HFD group (Fig. 3g). In contrast, HDCA, deoxycholic acid (DCA), and β-HDCA levels in the control and ST treatment groups were higher than in the HFD group (Fig. 3g). The data suggest that ST treatment may be implicated in BA metabolism.

### FMT alters gut microbiota composition in HFD-fed rats

**Fig. 4:**
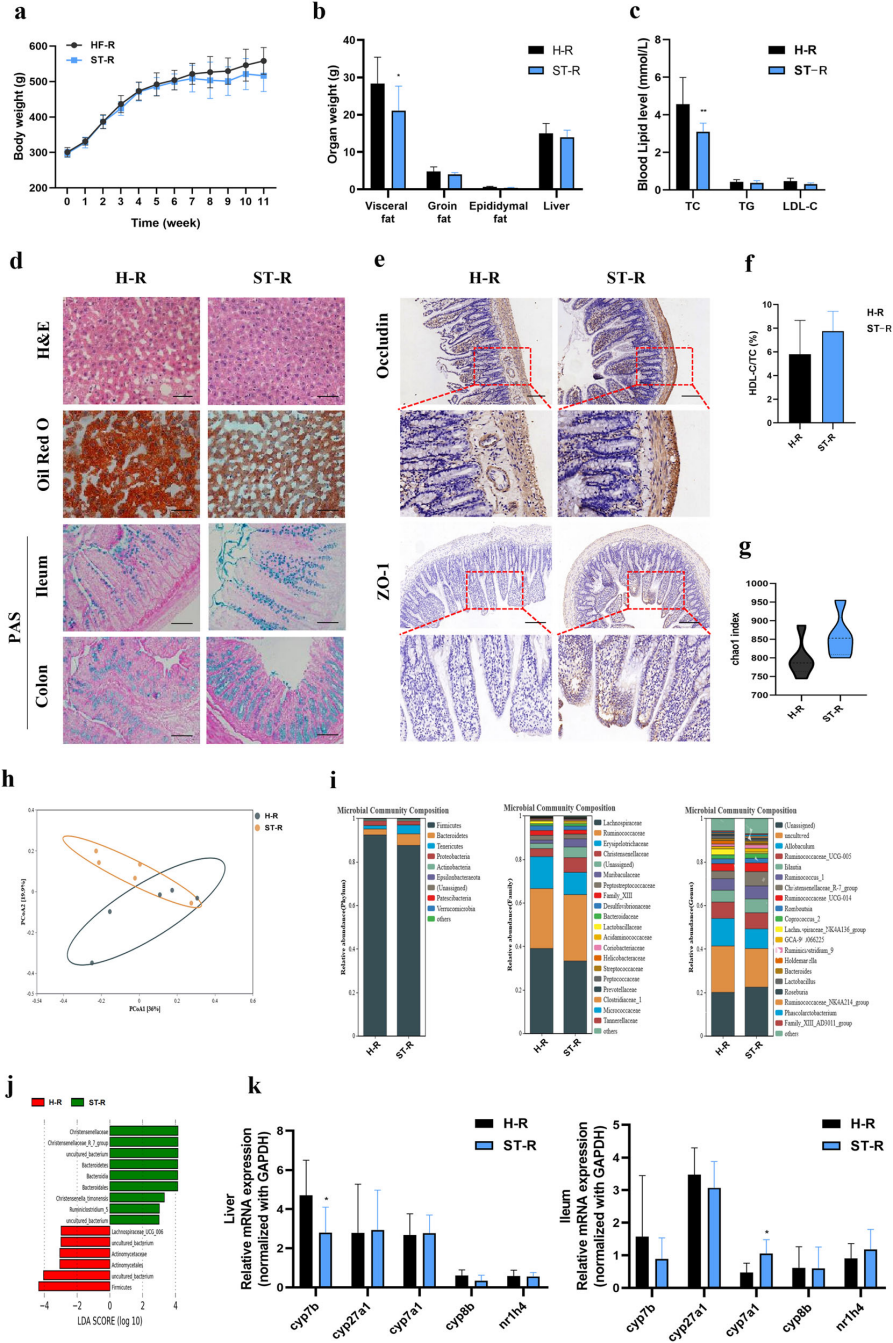
FMT improved the hepatic steatosis, intestinal barrier, and altered gut microbiota in HFD fed rats. **a** Changes in the body weight of rats over 11 weeks; **b** organ weights of rats in the end of the experiment; **c** serum levels of lipid (TC, TG, LDL-C); **d** histopathological staining of liver, ileum, and colon (H&E, ORO, and AB-PAS), Scale bar as above; **e** immunohistochemistry of Occludin and ZO-1 protein in ileum, Scale bar: 200 μm; **f** the ratio of HDL-C/TC in serum; **g** the α-diversity index (chao1) of gut microbiota; **h** the β-diversity analysis (PCoA) of gut microbiota; **i** relative abundance of gut microbiota in the phylum, family, and genus level; **j** linear discriminant analysis Effect Size (LEfSe) between H-R and ST-R group; **k** BAs metabolism-related gene expression in liver of rats; **l** BAs metabolism-related gene expression in ileum of rats. Data are expressed as mean±standard deviation. *n* = 6. One-way ANOVA was used to analyze statistical differences; compared with control group, ^#^*P* < 0.05, ^##^*P* < 0.01; compared with HFD group, ^*^P < 0.05, ^**^P < 0.01.

To further elucidate the mechanism(s) behind the ST-mediated improvement in HFD-induced hyperlipidemia, we performed FMT on ATB treated rats that had been reared in specific pathogen-free conditions. Here, we used stools from HFD-fed rats with and without ST treatment. Following FMT, the body weight of rats in the ST-R group decreased by the end of the experiment but this was not statistically significant (Fig. 4a). Visceral fat weight and the serum total cholesterol levels in the ST-R group were significantly lower than the H-R group (Fig. 4b, c). The HDL-C/total cholesterol ratio in the ST-R group was higher than in the H-R group, but again, this was not statistically significant (Fig. 4f).

Obvious lipid droplets were observed in histological liver sections in H-R rats with less observed in ST-R rats (Fig. 4d). Consistent with the above results, the number of goblet cells in the ileum and in the colon of ST-R rats were significantly higher compared to the H-R group. Further, compared to the H-R group, ST-R also increased the expression of Occludin and ZO-1 in the ileum (Fig. 4e). Analysis of the intestinal microflora showed that the overall structure of the intestinal microflora was slightly different in the ST-R group compared to the H-R group and compared to H-R group, the α-diversity of the ST-R group was significantly increased as measured by the Chao 1 index (Fig. 4g). The relative abundance of *Bacteroidetes* and *Tenericutes* at the phylum level was upregulated in rats that had received a FMT from ST rats. Compared to the H-R group, the ST-R group showed a significant increase in the relative abundance of *Bacteroidetes* and *Tenericutes* by 2.16-, and 2.85- fold, respectively. At the family level, compared with H-R group, the relative abundance of *Erysipelotrichaceae* in the ST-R group was significantly reduced by 0.77-fold. Based on Linear discriminant analysis Effect Size (LEfSe), species with a significantly different abundance (LDA score > 2), including *Firmicutes*, *Actinomycetales*, *Actinomycetaceae*, and *Lachnospiraceae_UCG006*, are biomarkers in the H-R group. In contrast, *Christensenellaceae*, *Christensenellaceae_R_7_group*, *Bacteriodetes*, *Bacteroidia*, *Bacteroidales*, *Christensenella_timonensis*, and *Ruminiclostridium* are biomarkers in the ST-R group (Fig. 4j). In addition, ST-R rats exhibited elevated iliac *cyp7a1* and reduced hepatic *cyp7b* mRNA expression (*P* < 0.05) (Fig. 4k). These results suggest that fecal transplants from the ST group may partially prevent HFD-induced liver steatosis and intestinal barrier damage. However, these data also suggest that these fecal transplants does not play a leading role in preventing hyperlipidemia and liver steatosis.

### ST increases BA excretion by upregulating CYP7A1 expression in HFD-fed rats

**Fig. 5:**
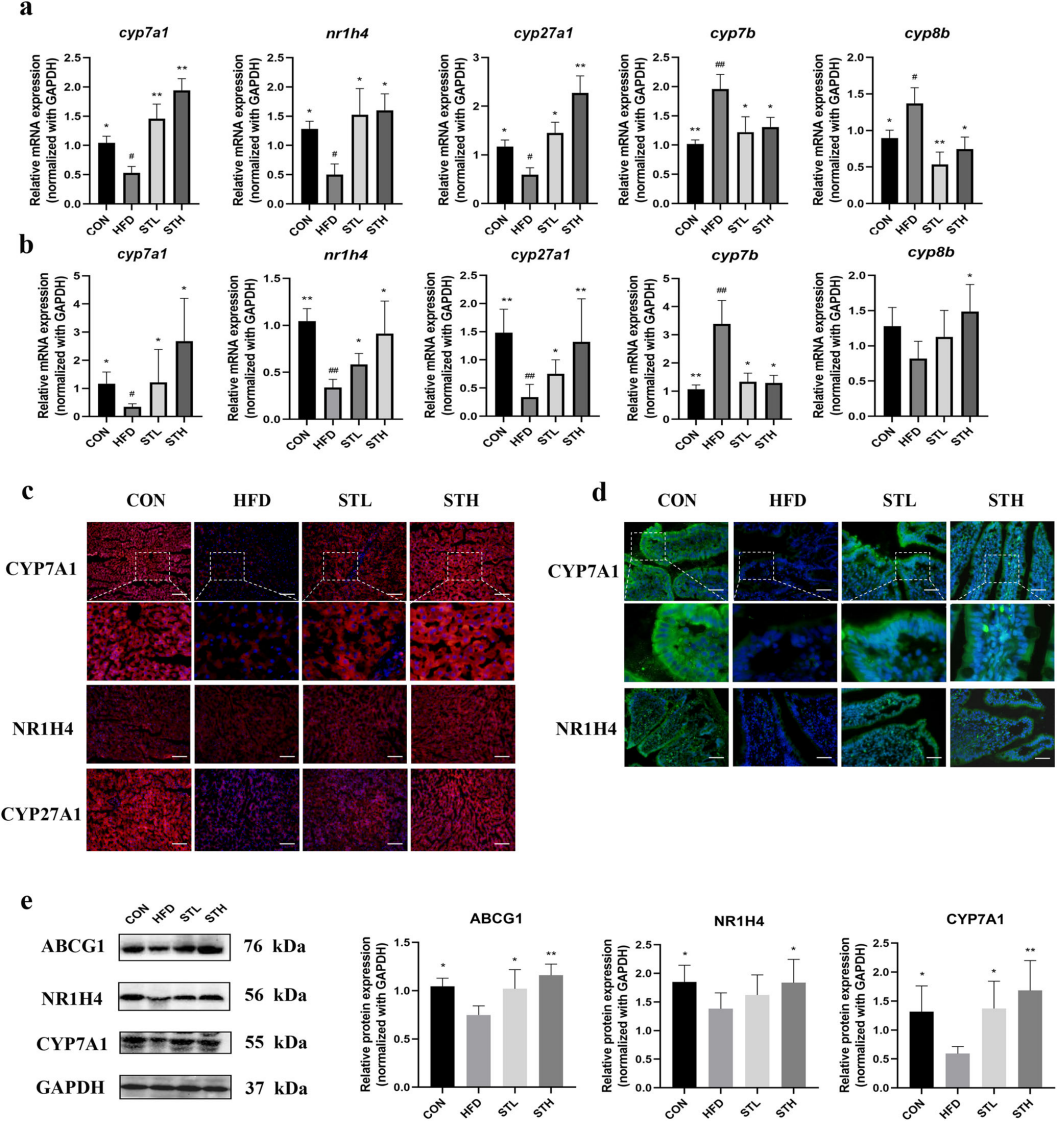
ST regulated BAs metabolism-related gene and protein expression levels in HFD-fed rats. **a** BAs metabolism-related gene expression in liver of rats; **b** BAs metabolism-related gene expression in ileum of rats; **c** immunofluorescence of pivotal protein expressions in liver of rats; **d** immunofluorescence of pivotal protein expression in ileum of rats; **e** BAs metabolism-related protein expressions in liver of rats. Data are expressed as mean±standard deviation. One-way ANOVA was used to analyze statistical differences; compared with control group, ^#^*P* < 0.05, ^##^*P* < 0.01; compared with HFD group, ^*^P < 0.05, ^**^P < 0.01.

To further study the metabolic pathway of BAs in liver–intestine circulation, we analyzed the expression of genes and proteins in HFD-fed rats. The results showed that *cyp7a1* mRNA expression in the liver and ileum of ST group were significantly higher than those in the HFD groups (*P* < 0.01). Compared to the control group, the HFD significantly reduced *nr1h4* and *cyp27a1* mRNA expression in the liver, and increased *cyp7b* and *cyp8b* expression (*P* < 0.05; *P* < 0.05; *P* < 0.01; *P* < 0.05, respectively). Compared to the HFD group, high-dose ST treatment significantly increased *nr1h4* and *cyp27a1* expression in the liver while it also decreased *cyp7b* and *cyp8b* expression by 3.64-, 3.83-, 0.67-, and 0.54- fold (*P* < 0.05), respectively (Fig. 5a, b). Except for *cyp8b, nr1h4*, *cyp27a1*, and *cyp7b* expression in the ileum was consistent with the trend among the groups in liver. We also performed IF staining and found that the expression of liver and ileum CYP7A1 and CYP27A1 was downregulated in the HFD group and upregulated in the ST groups (Fig. 5c, d). These data were consistent with our reverse transcription polymerase chain reaction (RT-qPCR) results. Interestingly, NR1H4 protein expression in the liver was not significantly different between the ST-treated group and the other groups, thereby suggesting that other factors may influence the protein expression of factors involved in bile metabolism. Our western blotting data agreed with the IF staining data (Fig. 5e). The expression of the adenosine triphosphate binding cassette transporter G1 (ABCG1) protein was significantly downregulated in the HFD group and upregulated in ST groups (*P* < 0.05) (Fig. 5e).

### CDCA combined ST ameliorates hyperlipemia and alters the gut microbiota in HFD-fed rats

**Fig. 6:**
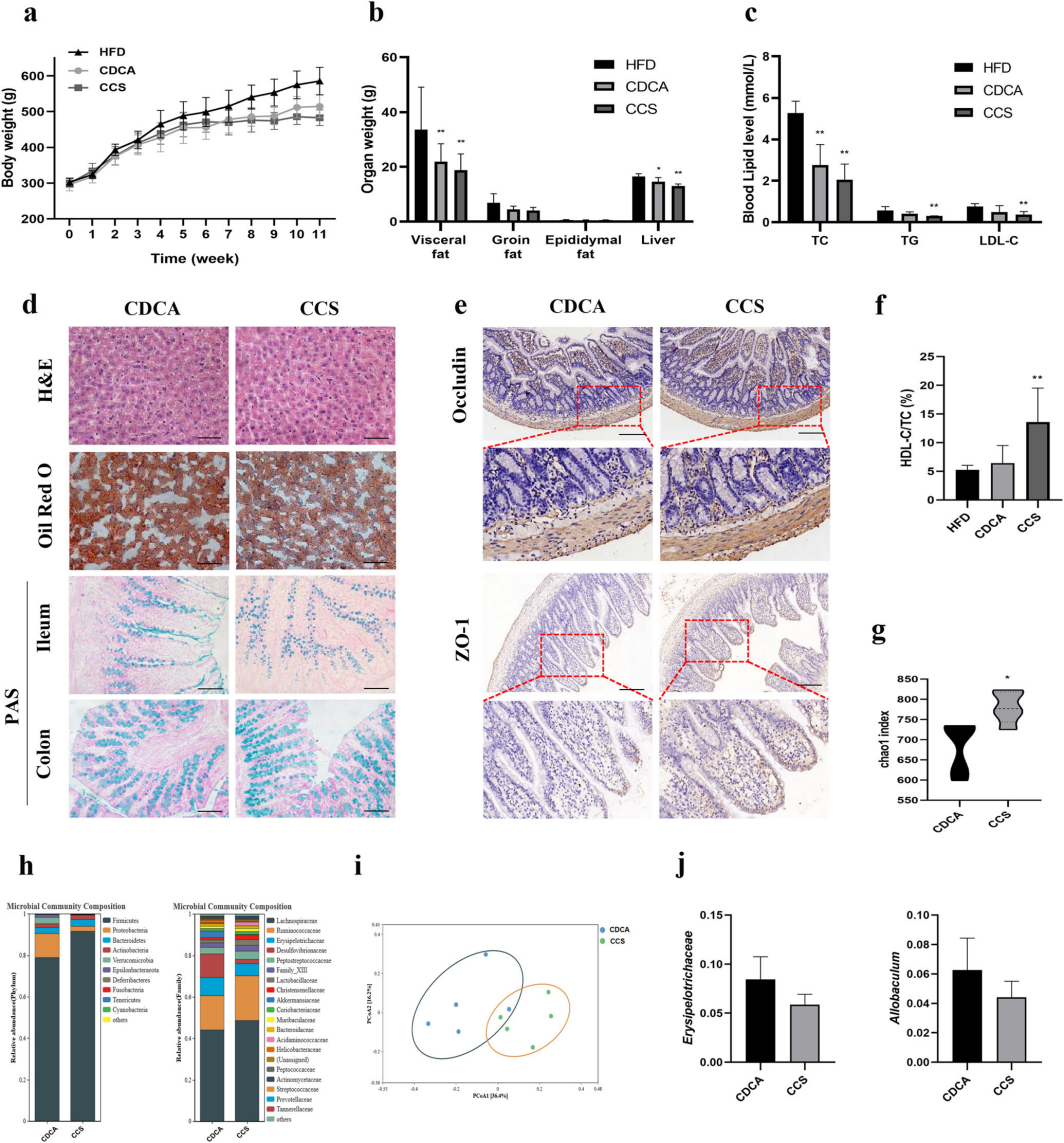
CCS improved the hepatic steatosis, intestinal barrier, and altered gut microbiota in HFD fed rats. **a** Changes in the body weight of rats over 11 weeks; **b** organ weights of rats in the end of the experiment; **c** serum levels of lipid (TC, TG, and LDL-C); **d** histopathological staining of liver, ileum, and colon (H&E, ORO, and AB-PAS), Scale bar as above; **e** immunohistochemistry of Occludin and ZO-1 protein in ileum, Scale bar: 200 μm; **f** the ratio of HDL-C/TC in serum; **g** the α-diversity index (chao1) of gut microbiota; **h** relative abundance of gut microbiota in the phylum and family level; **i** the β-diversity analysis (PCoA) of gut microbiota; (**j**) relative abundance of biomarker genra in the family and genus level. Data are expressed as mean±standard deviation. One-way ANOVA was used to analyze statistical differences; compared with control group, ^#^*P* < 0.05, ^##^*P* < 0.01; compared with HFD group, ^*^P < 0.05, ^**^P < 0.01.

In non-alcoholic fatty liver disease (NAFLD) and other obesity-related diseases, there is increasing evidence demonstrating the involvement of BAs^22^. Rats were fed an HFD for 11 weeks, and treated with CDCA or CDCA combined ST (CCS) from the 8th to the 11th week. Both groups exhibited the similar increase in body weight (Fig. 6a). In the CDCA and CCS groups, visceral and liver weights were significantly reduced (*P* < 0.05) (Fig. 6b). Levels of serum total cholesterol, triglyceride, and LDL-C were lower in the CCS group, and the ratio of HDL-C:total cholesterol was higher (Fig. 6c, f). Further, we observed reduced vacuolation of hepatocytes and lipid load of liver cells in the CCS group compared to the CDCA group (Fig. 6d). The number of goblet cells in the ileum and colon of both groups were upregulated. Based on the statistical results of OTU, we determined the α diversity of the samples. The chao 1 index is shown in Fig. 6g. Compared to the CDCA group, CCS increased the expression of Occludin and ZO-1 in the ileum using IHC (Fig. 6e). The β-diversity of the gut microbiota in the two groups is shown in the PCoA analysis in Fig. 6h. Compared to the CDCA group, the relative abundance of *Proteobacteria* at the phylum level, *Ruminococcaceae* at the family level, and *Allobaculum* and *Roseburia* at the genus level were decreased in the CCS group. In comparison, the relative abundance of *Lachnospiraceae* and *Erysipelotrichaceae* at the family level, *Ruminococcaceae_UCG-005*, and *Ruminococcaceae_UCG-014* were increased in the CCS group. Therefore, the anti-hepatic steatosis effects of CCS therapy and the changes in the intestinal flora can be reproduced in HFD-fed rats.

### CCS treatment upregulates the expression of CYP7A1 in HFD-fed rats

**Fig. 7:**
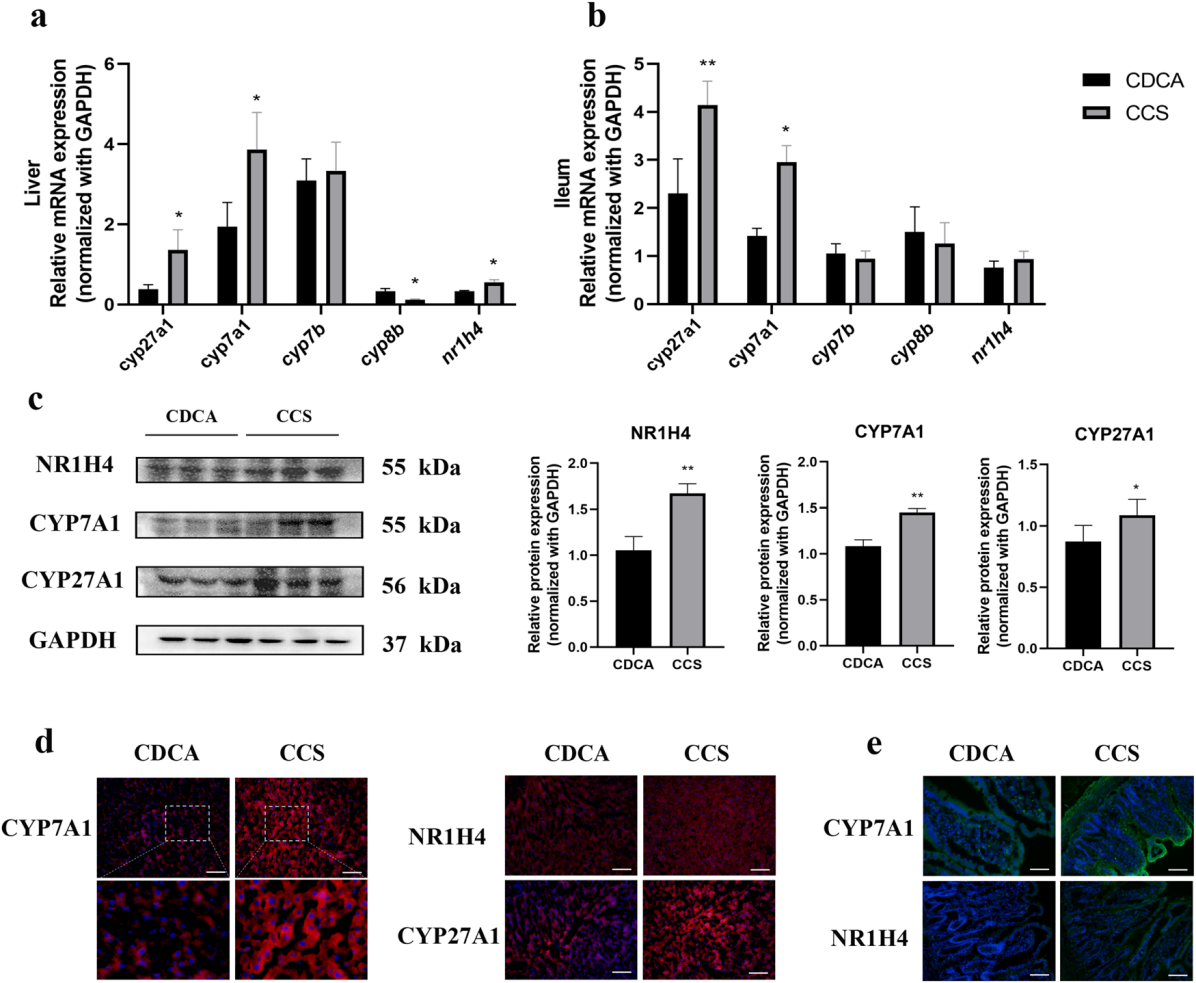
CCS regulated BAs metabolism-related gene and protein expression levels in HFD-fed rats. **a** BAs metabolism-related gene expression in liver of rats; **b** BAs metabolism-related gene expression in ileum of rats; **c** BAs metabolism-related protein expressions in liver of rats; **d** immunofluorescence of pivotal protein expressions in liver of rats; **e** immunofluorescence of pivotal protein expressions in ileum of rats. Data are expressed as mean ± standard deviation. One-way ANOVA was used to analyze statistical differences; compared with control group, ^#^*P* < 0.05, ^##^*P* < 0.01; compared with HFD group, ^*^P < 0.05, ^**^P < 0.01.

To explore the potential mechanism(s) behind the effects of combined CDCA/ST treatment on BA metabolism, we used RT-qPCR and western blotting to determine the expression of related mRNA transcripts and proteins. We found that CCS treatment upregulated hepatic mRNA expression of some transcripts (i.e., *cyp27a1*, 3.58-fold; *cyp7a1*, 1.99-fold; and *nr1h4*, 1.67-fold; *P* < 0.05) and downregulated *cyp8b* expression by 0.37-fold compared to the CDCA group. We also observed an increase in the iliac levels of *cyp27a1* (1.80-fold, *P* < 0.01) and *cyp7a1* (2.08-fold, *P* < 0.05) in the CCS group compared to the CDCA group. In Fig. 7c, western blotting data showed that compared to the CDCA group, CCS treatment significantly increased the expression of NR1H4, CYP7A1, and CYP27A1 in the liver (*P* < 0.01, *P* < 0.01, and *P* < 0.05, respectively). IF staining of liver and ileum samples were consistent with these data (Fig. 7d, e).

## Discussion

ST is known to improve obesity and related metabolic disorders^17^. However, the mechanisms by which it achieves these effects have, until now, remained unclear. In this study, we have shown that ST significantly alleviates HFD-induced hyperlipidemia and hepatic fat deposition, which are mainly dependent on the BA metabolic pathway. It should be noted that ST also improved the intestinal barrier function as well as the gut microbiota in HFD-fed rats resulting in a change in BA metabolism. We also found that BA profiles in the serum and feces of HFD-fed rats were significantly altered by ST treatment. Moreover, our data suggest that combined CDCA/ST treatment enhances cholesterol reduction by increasing cholesterol excretion in the liver.

Reverse cholesterol transport (RCT) reduces cholesterol by converting it into BAs^23^. During RCT in non-hepatic peripheral tissues, cholesterol is transferred to high density lipoprotein (HDL) particles and returned to the liver. Most of this cholesterol is then converted into BAs by the rate-limiting enzyme CYP7A1, which are then secreted into the small intestines where they promote absorption of dietary lipids^24,25^. Intestinal-hepatic circulation of BAs is an extremely effective process.

An estimated 90% of BAs are reabsorbed from the intestine, returned to the liver through the portal vein system, and secreted into the bile again. Less than 10% of intestinal BAs escape reabsorption to become substrates of the intestinal microbiota, before elimination in the feces^26,27^. In the present study, we found that, compared to the HFD group, BA content in the feces and serum of the ST group changed significantly. This suggests that ST treatment may alter the main enzymes involved in the BA biosynthetic pathway. Importantly, these enzymes may be involved in the production and secretion of primary BAs in the enterohepatic circulation. In the classic pathway of BAs biosynthesis, cholesterol is converted into 7α-hydroxycholesterol by CYP7A1 and in subsequent steps, cytochrome P450 Family 8 Subfamily B Member (CYP8B) and sterol 27-hydroxylase (CYP27A1) are required to synthesize CA^28^. Without CYP8B, the product is CDCA, which is formed via the activity of CYP27A1 alone. The alternative pathway is initiated by CYP27A1 and relies on 25-hydroxycholesterol 7-alpha-hydroxylase (CYP7B) to produce CDCA. In this study, we found that the hepatic mRNA and protein expression of CYP7A1 were significantly higher in the ST-treated group compared to the HFD group. We also found that *cyp27a1* expression was upregulated in the while *cyp7b* and *cyp8b* were downregulated in the rat liver. Furthermore, our data show that ST treatment leads to a significant increase in serum CDCA compared to the control HFD group. ST has been thought to promote the transformation of cholesterol to primary BAs, especially the production of CDCA, leading to a decrease in cholesterol deposition in the liver. As CDCA is an agonist of FXR, this may explain the significant increase of FXR expression in the liver and ileum in the ST group. In addition, ST treatment reduced serum TCA content, and we found that TCA is implicated in the development of liver cirrhosis^29^. Compared to the HFD group, ST treatment also led to a decrease in the serum levels of the FXR nuclear receptor antagonist, T-β-MCA. The increase of T-β-MCA level inhibited the FXR signal transduction in the intestine in a human metabolomic study and in *vitro* experiments^30^.

In the RCT, macrophages in the arterial wall that contain cholesterol transport cholesterol to apoA-I without any lipids through the ABCA1 transporter, forming new HDL particles. Further restriction of new HDL particles occurs through ABCG1, after which it is converted into mature HDL particles through the specific cholesterol efflux absorption of scavenger receptor type B. Importantly, ST treatment increased ABCG1 protein expression in the liver, indicating that ST may be involved in the regulation of cholesterol efflux in liver.

CDCA has been shown to regulate the metabolic function of the body by alleviating obesity and hyperglycemia caused by a HFD, activating brown adipose tissue, and by improving insulin resistance^31–33^. To further explore the possible mechanism of ST-mediated regulation of BA metabolism, we evaluated the therapeutic effects of CDCA with or without ST treatment on the cholesterol metabolism and intestinal barrier function under HFD feeding conditions. The results showed that ST improved the anti-hyperlipidemia effects of CDCA, and increased mRNA and protein expression of FXR and CYP7A1 in the liver. ST also improved metabolism of cholesterol by activating the CYP7A1 signaling pathway, suggesting that FXR and CYP7A1 may be involved in ST-mediated cholesterol metabolism and BA biosynthesis. Taken together, our results show that ST treatment improves HFD-induced cholesterol disorder by mediating cholesterol and BA metabolism.

BAs are necessary for cholesterol metabolism and for the intestinal environment. The observed changes in the intestinal environment are likely related to ST, but little is known about the host factors that induce changes in the intestinal barrier. In this study, we found that ST improves intestinal barrier function and changes the composition of the intestinal microflora. Impairment of the intestinal barrier, especially a decrease in intestinal paracellular permeability and the expression of tight junction genes and proteins, was observed in mice and rats fed on an HFD^34,35^. These changes in the intestinal barrier allows endotoxins to enter the circulatory system to further activate the inflammatory pathway^36^. A thin layer of colonic mucus associated with increased permeability is also observed in animal models of experimental colitis. This phenotype allows bacteria to gain direct contact with the mucosa^37^. Consistent with these studies, we observed a larger number of goblet cells in the ileum and colon of the ST treatment group, and increased expression of tight junction proteins in the intestine. Therefore, we hypothesize that ST treatment generated a stronger intestinal barrier which may prevent invasion of the bacterial lipopolysaccharide into the ileum and colon, thereby preventing differentiation of macrophages into the pro-inflammatory macrophage M1^38^.

Previous studies have shown that in rodents fed on an HFD, more goblet cells in the colon were associated with reduced obesity and inflammation^39,40^. Small intestinal expression of occludin, antimicrobial REG III/γ, and IL-22 in HFD mice has been shown to decrease significantly with a concurrent and significant increase in intestinal permeability^11^. Therefore, we believe that ST may prevent HFD-induced damage induced by strengthening the intestinal barrier via mucus-producing cells. In addition, in rats fed with a HFD, FMT from ST-treated rats showed similar protective effects on the intestinal barrier compared to ST-treated rats, thereby suggesting the involvement of the intestinal microflora in this protective phenotype.

There is growing concern that HFD has changed the genetic composition and metabolic activity of the human microbiota^41^. Given the key role of the intestinal microflora in cholesterol metabolism, it may be necessary to induce a change in microflora composition in clinical practice to generate healthier conditions. Therefore, focus on the modulatory effects against symbiosis and activation of the gut-liver axis is warranted. In our study, ST treatment resulted in a significant decrease in the abundance of *Erysipelotrichaceae* at the family level and *Allobaculum* at the genus level. Overgrowth of *Erysipelotrichaceae* is related to NAFLD progression, and the number of obese subjects increased in HFD-induced obesity in mice^42^. The abundance of *Allobaculum* is significantly correlated with serum and liver lipids, and carbohydrate profiles^43^. In addition, the HFD also increased the abundance of *Peptostreptococcaceae* at the family level, which are implicated in atherosclerosis^44^. This change was reversed by ST treatment. However, the mechanism behind ST-mediated regulation of the intestinal microflora and BA metabolism is still not clear. Therefore, we performed FMTs to determine whether the donor group also transferred the disease or health-related phenotype. The results showed that the FMT from the ST group promoted changes in the intestinal microflora and prevented the accumulation of lipids in the liver.

In this study, FMT altered the composition of the gut microbiota in the ST-R group, including *Christensenellaceae, Bacteriodetes*, *Bacteroidia*, *Bacteroidales*, *Christensenella_timonensis*, and *Ruminiclostridium*. We show that one of the most abundant groups was the *Christensenellaceae* family. As the richness and diversity of microorganisms in the gut increases, there is a negative correlation between the pathological features of metabolic syndromes such as obesity, hypertriglyceridemia, and body mass index. Moreover, the decrease in lipid biosynthesis and energy metabolism in people with a high abundance of *Christensenellaceae*, may explain the negative relationship between body weight and *Christensenellaceae*^45,46^. Changes in the relative abundance of *Bacteroidia*, which belongs to *Bacteroidetes*, is negatively correlated with the obesity phenotype, which is consistent with previous studies^47,48^. Gut-dwelling bacteria of the *Christensenellaceae* family are thought to act as keystones of the human gut ecosystem to prevent adipogenesis^49^. In the H-R group, *Actinomycetales* of the *Actinomycetaceae* family is associated with Type 2 diabetes and liver steatosis^50^. Baseline levels of *Lachnoclostridium* and *Actinomyces* were positively correlated with hepatic steatosis^51^. To summarize, FMT improved the intestinal microflora and intestinal barrier function, thus providing a better environment for the synthesis and metabolism of BAs, leading to improved intestinal-hepatic circulation.

The protective effects of FMT against obesity and other related diseases, including NAFLD, are not yet fully understood. Some researchers have hypothesized that production of antibacterial substances, enhancement of epithelial barrier function, and regulation of the immune system and subsequent intestinal inflammation may be implicated^52^. However, ST-R group did not display the same therapeutic effects as well as the ST treatment groups. Therefore, ST treatment may involve other factors that warrant further investigation. These findings may provide an explanation for ST-mediated improvements in cholesterol metabolism and liver fat accumulation across many different physiological systems (e.g., BA biosynthesis and intestinal environment).

This study provides novel insights that suggest that the intestinal barrier may be an important target organ for lipid reduction. Furthermore, CCS treatment may provide additional therapeutic benefit in HFD-induced hepatic steatosis and weakening of the intestinal barrier compared to CDCA treatment alone. Moreover, our data prove that specific gut microbiota species, such as *Erysipelotrichaceae* and *Allobaculum* may be involved in the metabolism of BAs and cholesterol. Dietary ST supplements in suitable amounts may potentially induce health benefits for the host and is, therefore, a promising strategy for clinical management of obesity-associated disorders.

Taken together, ST treatment reduces fat accumulation in the liver and improves intestinal barrier function. ST also enriches serum levels of CDCA and activates the CYP7A1 pathways, leading to the regulation of BA and cholesterol metabolism. CDCA/ST combination treatment may amplify the beneficial effects by further lowering cholesterol and hepatic steatosis. The results from this study suggest a simple and promising avenue for lowering lipid accumulation. Nonetheless, future studies are needed to further explore the precise mechanisms of these effects on the gut-liver axis.

## Methods

### Animal study I

All animal experiments were conducted in accordance with the National Institutes of Health guidelines for the care and use of experimental animals and approved by the Ethics Committee of Southern Medical University (Guangzhou, China). Forty SD rats (Specific pathogen-free grade, male, eight-week-old) were purchased from Southern Medical University Laboratory Animal Co. Ltd. (Guangzhou, China). The SD rats were maintained under specific pathogen-free conditions with free food and water (room temperature at 22 ± 2 °C, humidity at 45 ± 5%, 12 h light/dark cycle). After acclimatization for a week, thirty-two rats were fed an high-fat diet (45% of calories derived from fat) for seven weeks. The other eight rats served as the control group, which fed a normal diet (10% of calories derived from fat) for seven weeks. Then, the rats fed on HFD were assigned to four groups randomly (*n* = 8) in a feeding experiment: (1) HFD group (fed on HFD); (2) HFD+low-dose ST group (fed on HFD and 100 mg/kg of ST administered intragastrically); (3) HFD+high-dose ST group (fed on HFD and 200 mg/kg of ST administered intragastrically); and (4) HFD+simvastatin group (fed on HFD and 5 mg/kg of simvastatin administered intragastrically). All interventions lasted 4 weeks. The bodyweight of rats was measured each week.

### Fecal microbiota transplantation (FMT)

The feces of the donor group (HFD and HFD+high-dose stigmasterol group of rats, a total of twelve rats, *n* = 6) were collected every day during the second to the fourth week of the invention experiment for FMT and further analysis. The fecal samples were stored at −80 °C until use.

The recipient rats (a total of twelve rats, *n* = 6) were fed an HFD (45% of calories derived from fat) as described above for seven weeks. According to the protocol of antibiotic-treated rats established in previous research^53,54^, the recipient rats were intragastrically administered with antibiotic (ATB) cocktail (Ampicillin, Metronidazole and Neomycin at 0.25 mg/day and Vancomycin at 0.125 mg/day) for 5 consecutive days. For FMT, the fecal samples daily collected from each group of donor rats were homogenized and suspended in 0.9% sterile saline. After standing for half an hour, the supernatant of the mixture were taken. Each recipient rat received 0.4 mL/100 g body weight of respectively bacterial suspension intragastrically, i.e., HFD → H-R group, HFD+STH → ST-R group, correspondingly. The FMT was performed daily and lasted for four weeks (Fig. 1). The bodyweight of rats was measured each week. The fecal samples of recipients groups were separated collected and stored at −80 °C for further study. The experimental design of FMT is presented in Supplementary Fig. 1.

### Collection of Samples

At the end of feeding experiments, all rats were sacrificed anesthetized by intraperitoneal injection of Pelltobarbitalum Natricum. Following this, blood samples collection were performed from the abdominal aorta. Serum samples were collected by centrifuging the blood at 3000 rpm for 15 min, and then the supernatants were stored at −80 °C. The liver, ileum, epididymis fat, and abdominal fat were collected, weighed and stored at −80 °C. Serum samples were prepared and the serum concentrations of total cholesterol (TC), triglyceride (TG), LDL-C, high-density lipoprotein cholesterol (HDL-C), Alanine aminotransferase (ALT), and Aspartate aminotransferase (AST) were measured using the corresponding assess kits.

### Histopathological analysis

The liver, colon and ileum tissue of each rats were collected for further histopathological analysis. The tissues were fixed in 10% paraformaldehyde and embedded in paraffin wax. Sections of 5 μm thickness were made, deparaffinized, dehydrated, and then stained with H&E. A portion of the tissues was fixed and embedded in optimal cutting temperature compound. Tissue cryosections of 7 μm were made as per the above method from transverse plane. The Oil Red O (ORO) and Alcian Blue-Periodic Acid-Schiff (AB-PAS) staining were performed according to the methods described by Zhang et al. and Yoo et al.^55,56^.

### Gut microbiota analysis

The methods for analyzing the diversity and taxonomic profiles of fecal samples profiles of rats were described in our previous study^55^. The main coordinate analysis (PCoA) method was used to analyze the overall structural changes of intestinal flora. As for the beta diversity analysis, bray_curtis dissimilarity was used to calculate the distance between samples and obtain the sample distance matrix in the study.

### LC-MS analysis of BAs

The serum and fecal samples of rats were extracted with methanol. Then put the samples at −20 °C to precipitated protein. The extracts were evaporated to dryness, reconstituted in 100 μl 50% methanol (V/V) for further LC-MS analysis.The sample extracts were analyzed using an LC-ESI-MS/MS system. The analytical conditions were as follows, HPLC: column, Waters ACQUITY UPLC HSS T3 C18 (100 mm × 2.1 mm i.d. 1.8 µm); solvent system, water with 0.01% acetic acid and 5 mmol ammonium acetate (A), acetonitrile with 0.01% acetic acid (B); The gradient was optimized at 5 to 40% B in 0.5 min, then increased to 50% B in 4 min, then increased to 75% B in 3 min, and then 75 to 95% in 2.5 min, washed with 95% B for 2 min, finally ramped back to 5% B (12–14 min); flow rate, 0.35 mL/min; temperature, 40 °C. The effluent was alternatively connected to an ESI-triple quadrupole-linear ion trap (QTRAP)-MS.

AB 6500+ QTRAP® LC-MS/MS System, equipped with an ESI Turbo Ion-Spray interface, operating in negative ion modes and controlled by Analyst 1.6.3 software (AB Sciex). The ESI source operation parameters were as follows: ion source, turbo spray; source temperature 550 °C; ion spray voltage (IS) −4500 V; curtain gas (CUR) were set at 35.0 psi. DP and CE for individual MRM transitions was done with further DP and CE optimization. A specific set of MRM transitions were monitored for each period according to the eluted within this period. BAs contents were detected by MetWare (http://www.metware.cn/) based on the AB Sciex QTRAP 6500 LC-MS/MS platform.

### Animal study II

Eighteen SD rats were purchased and maintained under conditions as described above. The rats were fed an high-fat diet (45% of calories derived from fat) for seven weeks and then assigned to three groups (*n* = 6) randomly in a feeding experiment: (1) HFD group; (2) HFD+CDCA group (fed on HFD and 100 mg/kg of CDCA administered intragastrically); (3) HFD+CDCA+high-dose ST group (fed on HFD and 100 mg/kg of CDCA, 200 mg/kg of ST administered intragastrically). All interventions lasted 4 weeks. The bodyweight of rats was measured each week.

### Reverse transcription quantitative polymerase chain reaction (RT qPCR)

According to the manufacturer’s instructions, total RNA was extracted from liver and ileum of the rats using Trizol reagent (Invitrogen, Carlsbad, CA, USA). The concentration and purity of RNA were measured with NanoDrop™ Lite (Thermo Fisher Scientific, Waltham, MA, USA). The total RNA was reverse transcribed into cDNA using a PrimeScript™ RT Reagent Kit with gDNA Eraser (Takara Bio Inc., Kusatsu, Japan) and cDNA was amplified using GoTaq® qPCR Master Mix (Promega, Madison, USA). The threshold cycle (Ct) was recorded by a Light Cycler® 96 System (Roche Applied Science, Penzberg, Germany), using the following reaction conditions: 95.0 °C for 10 min, 40 cycles at 95.0 °C, 5 s, 60 °C, 30 s. The fold changes in mRNA expression were calculated using the 2^−△△^CT method. Primers for *nr1h4*, *cyp7a1*, *cyp27a1*, *cyp7b*, *cyp8b*, and *gapdh* were synthesized by Sangon Biotech (Shanghai) Co., Ltd. and are shown in Table 1.

**Table 1:** Primer sequences for quantitative RT- qPCR amplification.

Name	Sequence 5′-3′
gapdh	F: AGGTCGGTGTGAACGGATTTG
	R: TGTAGACCATGTAGTTGAGGTCA
cyp7a	F: GGGATTGCTGTGGTAGTGAGC
	R: GGTATGGAATCAACCCGTTGTC
cyp27a	F: CCAGGCACAGGAGAGTACG
	R: GGGCAAGTGCAGCACATAG
cyp8b1	F: CCTCTGGACAAGGGTTTTGTG
	R: GCACCGTGAAGACATCCCC
cyp7b	F: TGAGGTTCTGAGGCTGTGC
	R: TGGAGGAAAGAGGGCTACAA
nr1h4	F: CGGCGGGAAGAATAAAAGGG
	R: CACTTCCTTAGCCGGCAATC

### Western blotting

The liver of rats was homogenized with radioimmunoprecipitation assay buffer and 1% Phenylmethysufonyl Fluoride. Then, 40 μg of proteins from individual samples were separated by 10% SDS-PAGE and transferred onto polyvinylidene difluoride membranes. The membrane was blocked with 5% bovine serum albumin (BSA) at room temperature for 2 h and incubated with primary antibody at 1:1000 at 4 °C overnight, followed by secondary antibody (1:5000) for 1 h at room temperature. The following primary antibodies were used in this study: anti-NR1H4 antibody (Bioss, bs-12867R; 1:1000); anti-CYP7A1 antibody (Bioss, bs-21430R, 1:1000); anti-CYP27A1 antibody (Abcam, ab126785, 1:1000); anti-ABCG1 antibody (Bioss, bs-23382R, 1:1000). Densitometric analysis of blots was performed with Image J software.

### Immunofluorescent staining

The frozen sections of the liver and ileum was fixed with 10% paraformaldehyde for 15 min. The sections were permeableised in 0.25% Triton X-100 and then blocked with 5% goat serum for 30 min. After incubation with primary antibody overnight at 4 °C and secondary antibody for 1 h at RT, the slides were washed thrice with PBS and then counterstained using DAPI. The following primary antibodies were used in this study: anti-NR1H4 antibody (Bioss, bs-12867R; 1:200); anti-CYP7A1 antibody (Bioss, bs-21430R, 1:200); and anti-CYP27A1 antibody (Abcam, ab126785, 1:200). The images were obtained by fluorescence microscopy.

### Immunohistochemistry

The tissue section of ileum was deparaffinised, rehydrated, subjected to sodium citrate acid buffer to boiling water bath for approximately 92–95 °C for 15 min, and allowed to cool naturally to RT. A peroxidase blocking step using a 3% H_2_O_2_ blocking solution was performed for 10 min to reduce non-specific background and then blocked with 5% BSA for 20 min at RT. After incubation with primary antibody (anti-Occludin, Bioss, bs-10011R, 1:200; anti-ZO-1, Proteintech, No. 21773–1-AP, 1:150) overnight at 4 °C and HRP-conjucted secondary antibody for 1 h at RT. The slides were incubated with the DAB substrate chromogen solution at RT for 2 to 5 min and then washed three times, and counterstained with Hematoxylin counterstaining. After dehydration and film sealing, images are obtained by an automatic scanner.

### Statistical analysis

The SPSS software (version 20.0, Chicago, Illinois, USA) and GraphPad Prism software (version 8.00, San Diego, California, USA) were used for statistical analysis. One-way analysis of variance (ANOVA) and Tukey test were used to determine significant differences. All data were expressed as mean ± standard deviation. A *P* value < 0.05 was considered statistically significant.

## Supplementary information

Supplementary Figure 1

## Data Availability

The data that support the findings of this study are available from the corresponding author upon reasonable request.
