# Supplementary figures and images for: Stigmasterol attenuates hepatic steatosis in rats by strengthening the intestinal barrier and improving bile acid metabolism
Source: NPJ Sci Food. 2022 Aug 27;6:38. doi: 10.1038/s41538-022-00156-0 (PMC9420112; doi:10.1038/s41538-022-00156-0)

Supplementary Figure 1

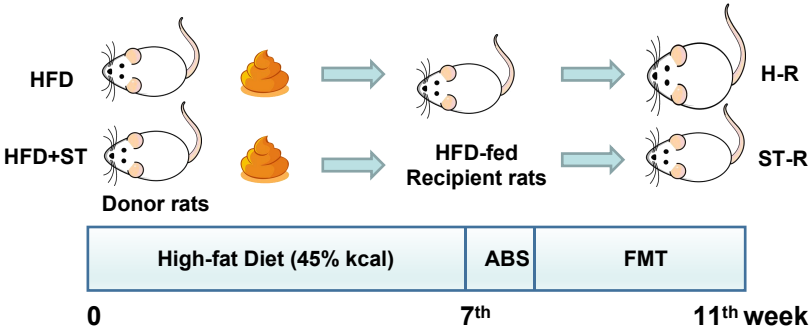

Supplement: Supplementary file 1 — Supplementary Figure 1 [file 41538_2022_156_MOESM1_ESM.pdf]
